# Mucin glycosylating enzyme GALNT2 suppresses malignancy in gastric adenocarcinoma by reducing MET phosphorylation

**DOI:** 10.18632/oncotarget.7081

**Published:** 2016-01-30

**Authors:** Shin-Yun Liu, Chia-Tung Shun, Kuan-Yu Hung, Hsueh-Fen Juan, Chia-Lang Hsu, Min-Chuan Huang, I-Rue Lai

**Affiliations:** ^1^ Graduate Institute of Anatomy and Cell Biology College of Medicine, National Taiwan University, Taipei, Taiwan; ^2^ Department of Pathology, National Taiwan University Hospital, Taipei, Taiwan; ^3^ Department of Internal Medicine, National Taiwan University Hospital Hsin-Chu Branch, Taipei, Taiwan; ^4^ Department of Surgery, National Taiwan University Hospital, Taipei, Taiwan; ^5^ Department of Life Science and Institute of Molecular and Cellular Biology, National Taiwan University, Taipei, Taiwan; ^6^ Research Center for Developmental Biology and Regenerative Medicine, National Taiwan University, Taipei, Taiwan

**Keywords:** gastric cancer, GALNT2, O-glycosylation, hepatocyte growth factor, receptor tyrosin kinase

## Abstract

Glycosylation affects malignancy in cancer. Here, we report that N- acetylgalactosaminyltransferase 2 (GALNT2), an enzyme that mediates the initial step of mucin type-O glycosylation, suppresses malignant phenotypes in gastric adenocarcinoma (GCA) by modifying MET (Hepatocyte growth factor receptor) activity. GALNT2 mRNA and protein were downregulated in GCAs, and this reduction was associated with more advanced disease stage and shorter recurrence-free survival. Suppressing GALNT2 expression in GCA cells increased cell growth, migration, and invasion *in vitro*, and tumor metastasis *in vivo*. GALNT2 knockdown enhanced phosphorylation of MET and decreased expression of the Tn antigen on MET. Inhibiting MET activity with PHA665752 decreased the malignant phenotypes caused by GALNT2 knockdown in GCA cells. Our results indicate that GALNT2 suppresses the malignant potential of GCA cells and provide novel insights into the significance of O-glycosylation in MET activity and GCA progression.

## INTRODUCTION

Glycosylation involves the attachment of a carbohydrate to a protein, lipid, or other organic compound, and mucin-type O-glycosylation is the most common. O-glycosylation in mammalian cells is initiated by the transfer of N-acetylgalactosamine from a sugar donor to serine or threonine hydroxyl residues, which is mediated by N-acetyl-galactosaminyltransferases (GALNTs). The resulting short glycan, a Tn antigen, is a cancer-associated carbohydrate structure [1]. There are 20 GALNT family members in humans, and the O-glycosylation they catalyze may affect the functional properties of secreted and membrane-bound proteins.

With the development of glycobiology, the expression and roles of GALNTs in malignancies, including gastric cancer, have been explored. For example, GALNT3 expression correlated with increased differentiation in gastric cancer and better prognoses [2]. GALNT2 knock-down *in vitro* was associated with increased cell proliferation, adhesion, and invasion [3]. Additionally, low intra-tumoral GALNT5 expression was detected in advanced stage gastric cancer patients with poor prognosis [4], and higher GALNT10 expression was found in diffuse type gastric cancers [5]. However, the mechanistic roles of GALNTs in gastric cancer progression remain unknown.

We have previously shown that dysregulation of GALNT2 contributes to the malignant progression of hepatocellular carcinoma and oral squamous carcinoma cells by modifying glycosylation of the epidermal growth factor receptor, a member of receptor tyrosine kinase (RTK) family [6,7]. In this study, we first examined the expression of GALNT2 in gastric cancer and its correlation with clinicopathological features. The effects of GALNT2 on gastric cancer cells and the underlying mechanisms were studied using *in vitro* and *in vivo* experiments.

## RESULTS

### GALNT2 expression is downregulated in human gastric carcinoma

**Figure 1:**
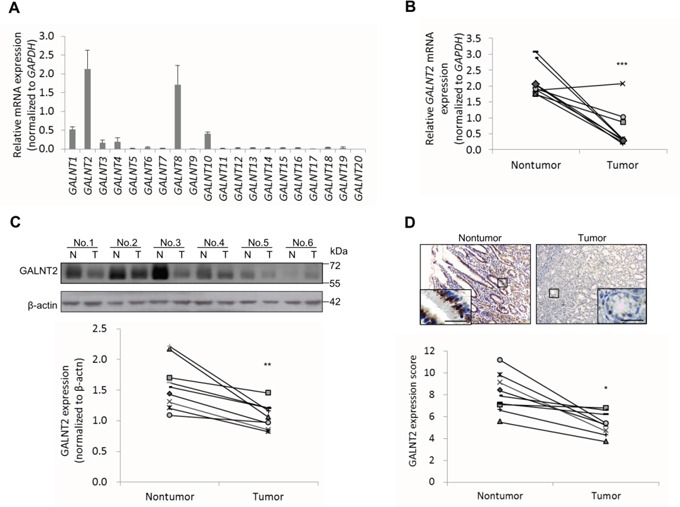
Expression of GALNT2 in human gastric cancers and non-tumorous mucosa **A.** The expression of *GALNT1-20*, as indicated, in non-tumorous stomach mucosa from GC patients (n=9) was analyzed by real-time RT-PCR. Relative *mRNA* levels were normalized to *GAPDH*. Results are expressed as mean ± SD for three independent experiments. **B.** *GALNT2* mRNA expression in pared non-tumorous mucosa and GC tissues (n=9) was analyzed. **C.** Expression of GALNT2 protein in GC and non-tumorous mucosa. Upper panel, representative Western blot. Lower panel, statistical analysis of GALNT2 expression in paired GC and non-tumorous mucosa (n=9). N, non-tumorous gastric mucosa; T, tumor tissue. **D.** Expression of GALNT2 in GC tissues was evaluated by immunohistochemistry (upper panel); quantitative results are shown in the lower panel (n=9). Scale bars, 20μm. (*p < 0.05; **p < 0.005; ***p < 0.001).

To investigate the potential role of GALNT family genes in gastric carcinoma (GC), we first analyzed the expression of *GALNT1-20* in non-cancerous gastric mucosa using real-time RT-PCR. Among the 20 genes, only *GALNT2* (2.1±0.5) and *GALNT8* (1.7±0.5) were highly expressed in non-cancerous gastric tissue (Figure 1A). We further analyzed GALNT2 expression in paired GC and non-cancerous tissues (n = 9). GALNT2 expression was significantly lower in GC tissues (0.6±0.6) than in their non-cancerous parts (2.1±0.5) (Figure 1B, ***p < 0.001). GALNT2 protein expression was also consistently lower in GC tissues in Western blotting (Figure 1C) and immunohistochemistry (Figure 1D) experiments (n=9).

**Figure 2:**
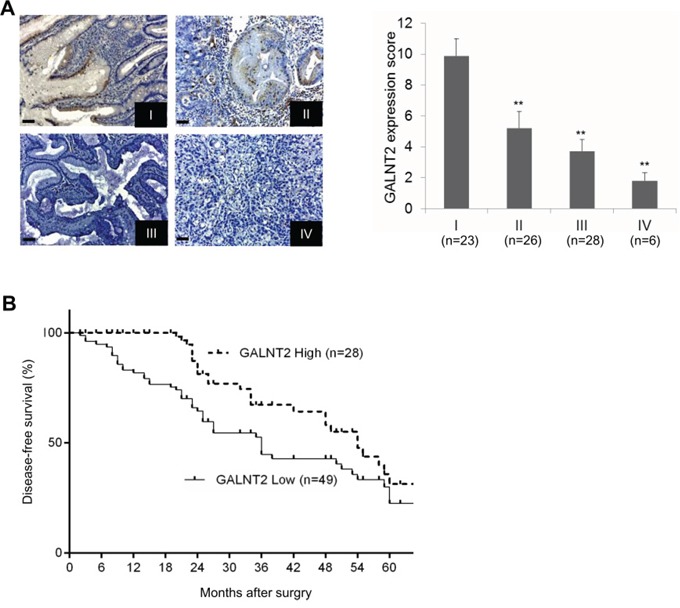
Relationships between GALNT2 expression and clinical features in GC **A.** Representative immunohistochemical staining of GALNT2 in GC tissues from stage I to IV (left panel), and semi-quantitative immunoreactivity scores of GALNT2 expression in stage I to IV gastric cancers. Scale bars, 20μm. **B.** Diseased-free survival of GC patients with high and low GALNT2 expression. (**p < 0.005).

To evaluate the role of GALNT2 expression in disease progression, immunohistochemical staining of GALNT2 was performed in GC tissues from 83 gastric cancer patients. GALNT2 staining intensity was scored using a semi-quantitative immunoreactivity scoring (IRS) system. Correlations between clinicopathological features and GALNT2 expression in gastric cancer are listed in Table 1. Low GALNT2 expression correlated with increased tumor depth, lymph node metastasis, and TNM stage. Additionally, GALNT2 expression was downregulated in more advanced gastric cancer (Figure 2A, **p < 0.005). Kaplan–Meier survival analyses showed that low GALNT2 expression correlated with shorter disease-free survival (DFS); the 3-year DFS was 25.8% for the high GALNT2 patient group and 18.2% for the low GALNT2 group (Figure 2B, *p* = 0.011). Collectively, these data revealed that GALNT2 expression is downregulated in advanced gastric adenocarcinoma and that reduced GALNT2 is associated with poorer prognosis.

**Table 1:** Clinicopathological correlation of GALNT2 expression in gastric cancers

Category/Number	GALNT2 expression		
	High (31)	Low (52)	*p*
**Age** (mean)	70.3±15.7	64.7±13.9	0.820
**Gender** (Male: Female)	16:15	32:20	0.910
**Lauren classification** Intestinal: Diffuse: Mixed	17: 12: 2	24:22:6	0.178
**Tumor depth** (T1: T2,3,4)	16:15	2:50	<0.001
**Lymph node metastasis** (Yes: No)	12:19	45:7	<0.001
**TNM Stages** (I+II : III+IV)	29:2	7:45	0.013
**3-year DFS** (%)	25.8	18.2	0.002

### GALNT2 knockdown in gastric cancer cell lines

**Figure 3:**
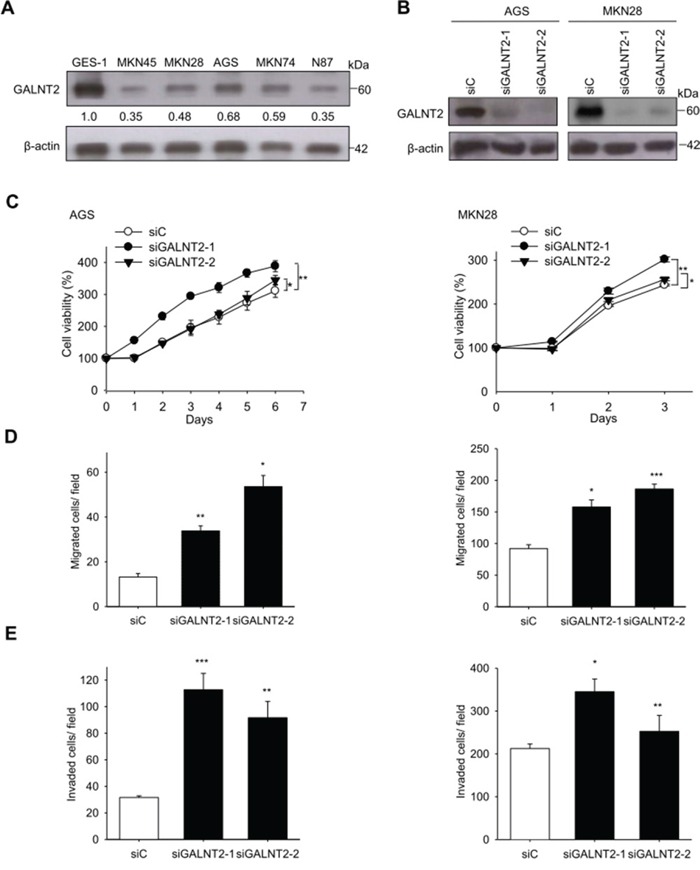
Effects of GALNT2 knockdown on malignant phenotypes in gastric cancer cells **A.** Expression of GALNT2 in five GC cell lines and GES-1 cells. GALNT2 protein expression was analyzed by Western blotting. **B.** Transfection of siRNAs targeting GALNT2 (siGALNT-1 and siGALNT-2) or control siRNA (siC) in AGS and MKN28 cells. The efficiency of GALNT2 knockdown was confirmed by Western blotting. **C.** Cell viability was analyzed by MTT assay at different time points. The results were graphed after standardization by siC (Day0) to 1.0 (Left, AGS cells; right, MKN28 cells). **D.** Effects of GALNT2 on cell migration in transwell migration assays (Left, AGS cells; right, MKN28 cells). **E.** Effects of GALNT2 on cell migration in matrigel invasion assays (Left, AGS cells; right, MKN28 cells). Data are presented as mean ± SD from 3 independent experiments. (*p < 0.05; **p < 0.005; ***p < 0.001).

To investigate the role of GALNT2 in gastric cancer, we first analyzed GALNT2 expression in 5 gastric cancer cell lines and in GES-1 cells using Western blotting. Among the 5 cancer cell lines, AGS and MKN28 cells expressed higher levels of GALNT2, whereas MKN45 cells expressed lower levels of GALNT2 (Figure 3A). We therefore chose AGS cells and MKN28 cells for siRNA GALNT2 knockdown experiments. GALNT2 knockdown was confirmed via Western blotting (Figure 3B).

### GALNT2 suppresses malignant phenotypes in AGS and MKN28 cells

To investigate the effects of GALNT2 on malignant phenotypes in gastric cancer, viability, migration, and invasion were measured in AGS and MKN28 cells with and without GALNT2 knockdown. The MTT assay showed that GALNT2 knockdown increased the viability of AGS and MKN 28 cells (Figure 3C). Migration and invasion were also markedly enhanced in AGS and MKN28 cells with GALNT2 knockdown (Figure 3D and 3E) (*p < 0.05, **p < 0.005, ***p < 0.001).

AGS cells and cells from the MKN45 gastric cancer line, which had relatively low GALNT2 expression, were transfected with pcDNA3.1(+)-GALNT2 plasmid to overexpress GALNT2. Successful ectopic GALNT2 expression was confirmed with Western blots (supplementary Figure 1A). Compared to the plasmid control, GALNT2 overexpression in AGS and MKN45 cells decreased viability (Supplementary Figure 1B), and inhibited cellular migration and invasion (Supplementary Figure 1C, Figure 1D) (*p < 0.05, **p < 0.005, ***p < 0.001). These *in vitro* experiments showed that GALNT2 downregulation might promote malignant progression in gastric cancer.

### GALNT2-knockdown increases tumor metastasis in nude mice

**Figure 4:**
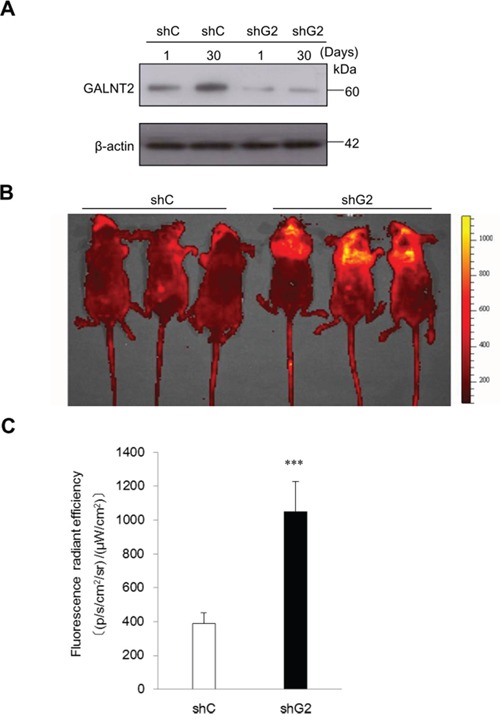
GALNT2 regulates gastric carcinoma cell metastasis in nude mice **A.** Western blot analysis of GALNT2 expression 1 and 30 days after transfection in control (shC) and GALNT2 knockdown (shG2) cells. β-actin was used as an internal control. **B.** Representative IVIS images of nude mice 30 days after infusion of gastric cancer cells via the tail vein. Markedly enhanced fluorescence signals were detected in the thoracic region of shG2 group. **C.** Quantitative determination of fluorescence signal intensities in shC and shG2 mice. (n=9 for each group, ***p < 0.001).

To investigate the effect of GALNT2 on tumor metastasis *in vivo*, nude mice were inoculated with shC- or shGALNT2-transfected AGS cells via the tail vein. The knockdown efficiency of shGALNT2 in AGS cells was confirmed by Western blot analysis (Figure 4A). IVIS images were used to monitor the status of tumor metastasis. As shown in Figure 4B and 4C, the fluorescence radiant efficiencies of the shGALNT2- transfected group (1051.9±176.8) were higher than those of the shC-transfected group (387.4± 66.0) 30 days after inoculation (Figure 4B & 4C). These results suggest that GALNT2 knockdown promotes gastric cancer metastasis *in vivo*.

### GALNT2 knockdown increased HGF-induced activation of MET and malignant potential in gastric cancer cells

**Figure 5:**
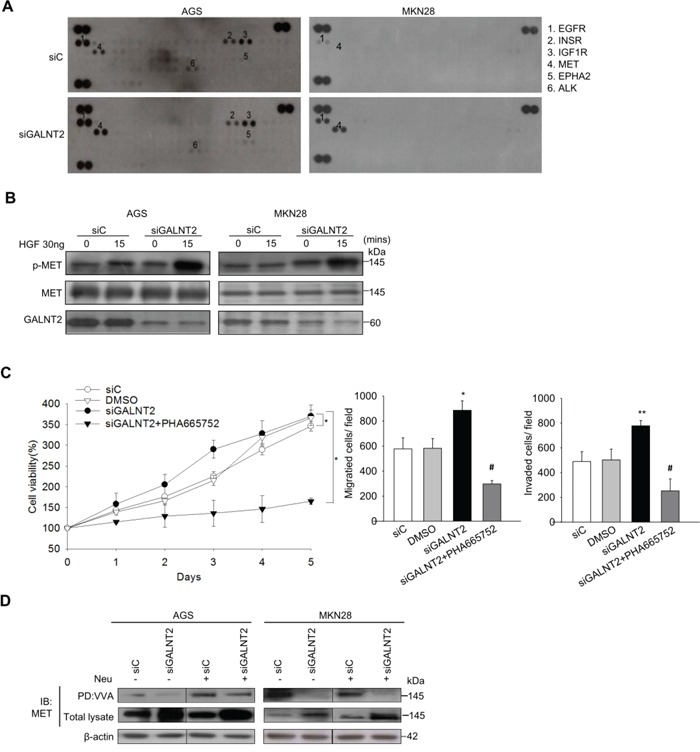
GALNT2 regulates MET activity in gastric cancer cells **A.** Human p-RTK array showing the effect of GALNT2 on RTK phosphorylation. Cell lysates of control and GALNT2 knockdown AGS or MKN28 cells were applied to p-RTK arrays. **B.** GALNT2 modulated HGF-induced phosphorylation of MET. Control and GALNT2-knockdown AGS and MKN28 cells were treated with HGF (30 ng/ml) for 15 minutes, and lysates were analyzed by Western blotting. **C.** The effect of the MET inhibitor PHA665752 on GALNT2-enhanced cell viability, migration, and invasion. AGS cells were treated with 3 μM PHA665752 and then analyzed. Data are presented as means ± SD from three independent experiments. **D.** GALTNT2 regulated O-glycosylation of MET. Some AGS and MKN28 lysates were treated with neuraminidase (Neu) to unmask the effects of sialyation, and all were then incubated with VVA-conjugated agarose beads. Proteins pulled down by VVA were analyzed by immunoblotting (IB) with anti-MET antibody. Knockdown of GALNT2 decreased VVA binding to MET in AGS and MKN28 cells. Total lysate was used as loading control. (*p < 0.05; **p < 0.005; ***p < 0.001; siGALNT2 versus siGALNT2+PHA665752; #*p* < 0.005).

Receptor tyrosine kinase (RTK) signaling is important for gastric cancer progression [18], and RTK activity is regulated by O-glycosylation [19]. To evaluate the effect of GALNT2 knockdown on RTK activity, a human phospho-RTK array including 49 different RTKs was performed. As shown in Figure 5A, GALNT2 knockdown markedly increased the phosphorylation of MET, EGFR, IGF1R, and EphA2 in AGS cells. Among them, the increase in MET phosphorylation was largest in AGS and MKN28 cells. We then validated the effect of GALNT2 knockdown on HGF-induced MET activation. Total cell lysates were immunoblotted with antibodies for MET p-Y1234/5 and total MET. As shown in Figure 5B, GALNT2 knockdown increased HGF-induced MET phosphorylation in AGS and MKN28 cells. Furthermore, treatment with PHA665752, a class of small molecules that inhibit MET enzymatic activity, reduced viability, migration, and invasion in siGLANT2-treated cells (Figure 5C). In contrast, GALNT2 overexpression in AGS and MKN28 cells reduced HGF-induced MET phosphorylation (Supplementary Figure 2). These results indicate that GALNT2 regulates malignant phenotypes in gastric cancer by modulating of MET activity.

### GALNT2 modulates o-glycosylation of MET

To investigate whether GALNT2 can modify O-glycans on MET, VVA lectin was used to detect Tn antigen (GalNAc-o-Ser/Thr) expression in the presence or absence of GALNT2 knockdown. To minimize the effects of sialic acids on lectin binding, neuramidase digestion of the cell lysate was performed. MET in control and GALNT2-knockdown AGS and MKN28 cells was immuno-precipitated. As shown in Figure 5D, GALNT2-knockdown reduced VVA binding to MET O-glycan, regardless of neuramidase treatment. These results indicated that GALNT2 modifies O-glycan on MET in gastric cancer cells.

### GALNT2 knockdown increased MET expression in gastric cancer

**Figure 6:**
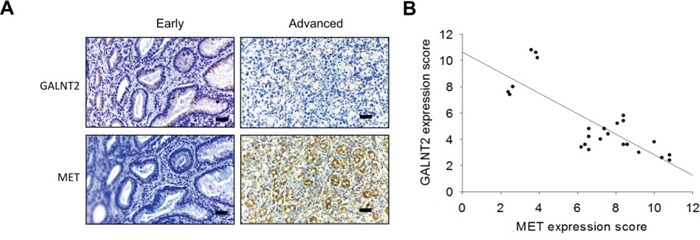
Correlation between GALNT2 and MET expression in GC tissues **A.** Representative immunohistochemistry (IHC) staining of GALNT2 (upper panel) and MET (lower panel) in early (left) and advanced (right) stage gastric cancer tissues. **B.** Linear regression of IHC scores of GALNT2 and MET in gastric cancer (n=24). The correlation coefficient (r) was −0.801; *p* < 0.001. Scale bars, 40μm.

GALNT2 knockdown in AGS and MKN28 cells was associated with increased total MET expression (Figure 5D and Supplementary Figure 3). IHC staining of GALNT2 and MET was performed in gastric cancer tissues (Figure 6A), and IHC scores for GALNT2 and MET staining were correlated using a linear regression model (Figure 6B). The Pearson's correlation coefficient between the GALNT2 and MET IHC scores was 0.801 (*p* < 0.001).

### GALNT2 knockdown affects gene expression

**Figure 7:**
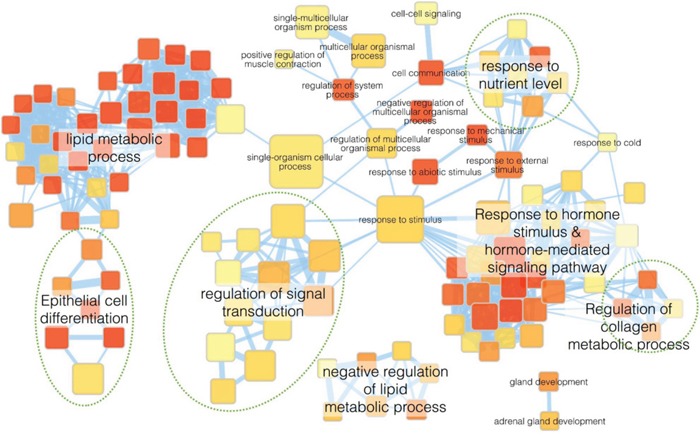
Functional maps of genes with altered expression after GALNT2 knockdown in AGS cells Enrichment results of *GALNT2* knockdown in AGS cells were mapped as networks. Nodes represent enriched gene sets (p < 0.05), and edges represent gene overlap scores between nodes above the threshold (0.6). Node color indicates enrichment p-value (red: low; yellow: high). Node size is proportional to the number of genes belonging to the corresponding gene set. Edge thickness is proportional to the overlap score. Groups of functionally related gene sets are highlighted in colors and labeled.

**Figure 8:**
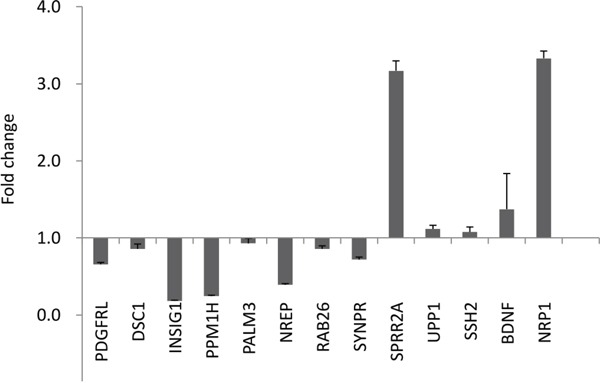
Quantitative RT-PCR validation of microarray results Altered expression (fold change) of selected genes upon *GALNT2* knockdown in AGS cells compared with control. Values greater than 1 represent gene upregulation and values less than 1 represent gene downregulation. Results were analyzed from three independent *GALNT2* knockdown AGS cell groups and their corresponding controls and are presented as mean ± SD.

To better understand the molecular mechanisms by which *GALNT2* gene expression affects gastric cancer progression, we evaluated global gene expression changes in control and *GALNT2-*knockdown AGS cells. All microarray experiments were performed in triplicate, with three hybridizations conducted for each group of *GALNT2* knockdown cells against the corresponding control. Functional enrichment and network analysis showed that *GALNT2* knockdown in AGS cells lead to differential gene expression in various pathways, including response to nutrient levels, response to hormone stimuli and hormone-mediated signaling pathways, regulation of collagen metabolic processes, regulation of signal transduction, and epithelial cell differentiation (Figure 7). The differential expressions (≥ 1.5-fold change, *p* < 0.05) of functionally related gene groups are provided in Supplementary Table S1. Genes selected for real-time RT-PCR validation are shown in Figure 8. GALNT2 suppression downregulated genes related to signal transduction regulation (PALM3 and NREP), and upregulated a gene that regulates epithelial cell differentiation (SPRR2A), in AGS cells.

## DISCUSSION

In this study, we show that GALNT2 is an important glycosylation enzyme in the human stomach. GALNT2 was downregulated in gastric adenocarcinoma, and lower GALNT2 expression correlated with more advanced tumor stage, lymph node metastasis, and reduced disease-free survival. GALNT2 downregulation promoted malignant phenotypes in gastric cancer, including cell proliferation, migration, invasion, and tumor metastasis, by increasing MET phosphorylation.

Altered glycosylation is a common feature of cancer. Tumor-associated carbohydrate antigens, such as Tn and T antigens, are associated with tumor progression. GalNAc-transferases (GALNTs) are crucial O-glycosyltransferases that initiate the formation of mucin-type O-glycan (Tn antigen) and are differentially expressed in various tissues. Whether GALNTs may act as markers of gastric cancer or potential targets for vaccines remains unclear. Here, we show that the GALNT1, 2, and 8 enzymes play important roles in human stomach tissue, and GLANT2 was the most highly expressed of these three. In our previous studies, downregulation of GALNT2 in hepatocellular carcinoma [6] and neuroblastoma [13] was associated with increased malignancy. Here, we showed that downregulation of GALNT2 also correlated with increased malignant progression in gastric cancer. Additionally, our *in vitro* results were similar to a previous study (3) in that GALNT2 knockdown was associated with increased cell proliferation, adhesion, and invasion in gastric cancer cells.

Receptor tyrosine kinases (RTKs) are promising targets for cancer treatment, since dysregulated RTK signaling pathways are important for malignant transformation [14]. Deng et al. showed that RTK/RAS genomic amplifications, including FGFR2, EGFR, Her2, and MET, occur in approximately 37% of gastric cancer patients [15]. GALNT2 can also modify the activity of EGFR [6] or the IGF-1 receptor [13] in cancer. Our p-RTK array results showed for the first time that GALNT2 regulates the phosphorylation of multiple RTKs in GC, especially Hepatocyte growth factor receptor (MET). A MET inhibitor, PHA665752, reversed the effects of GALNT2 knockdown on gastric cancer cell survival, invasion, and migration, suggesting that GALNT2 affects GC progression by modifying MET activity. MET is a pleiotropic RTK, and the MET gene is amplified in a small but prognosis-poor subgroup of gastric cancer patients [16]. We also found that GALNT2 downregulation was associated with MET overexpression in our patient cohort. The exact cause of increased MET expression is not known, but it is possible that GALNT2-induced alterations in O-glycosylation of RTKs might change their stabilities [12] or degradation rates [17].

Furthermore, we showed that GALNT2 can modify MET O-glycosylation in gastric cancer cells. GALNT initiates mucin-type O-glycosylation by adding α-GalNAc to serine/threonine residues in proteins, resulting in the formation of Tn antigens. GALNT2 knockdown in gastric cancer reduced Tn antigen expression on MET, as evidenced by reduced VVA binding (Figure 5D). Aberrant Tn antigen formation might interfere with extension and lead to truncated O-glycans, which might be partially responsible for the dysregulated MET activation.

Future studies will help to clarify the findings presented here. First, the signaling pathways leading to increased proliferation, invasion, and migration in GALNT-2 knockdown gastric cancer cells were not explored in our study. However, an examination of global gene expression showed that many functionally related genes associated with hormone-mediated signaling, signal transduction, response to nutrient levels, and epithelial cell differentiation were differentially expressed following GALNT2 suppression in AGS cells. Additionally, GALNT2 knockdown in SGC7901 cells increases MMP-2 and TGF-beta expression [3], suggesting that altered glycosylation might modulate the expression of metalloproteases and cytokines and in turn affect cell phenotypes. Further studies of the molecular mechanisms underlying the effects of GALNT2 in gastric cancer would be helpful. Second, increased phosphorylation of other RTKs, including EGFR, was also shown in GALNT2 knockdown gastric cancer cells. EGFR overexpression was observed in 27-44% of resected gastric cancer tissues and was often associated with poor prognosis [18,19,20]. Our previous studies showed that GALNT2 modulated EGFR activity and suppressed EGF-induced proliferation, migration, and invasion in hepatocellular carcinoma cells [6]. Whether GALNT2 also suppresses EGF-induced phenotypes in gastric cancer cells requires further study. Finally, microRNAs might target GALNTs in bladder cancer [21] and during osteoblast differentiation [22]. The upstream regulatory events controlling GALNT expression in gastric cancer remain to be explored.

In summary, GALNT2 is frequently downregulated in advanced gastric cancer, and lower GALNT2 expression is associated with poor disease-free survival. Knockdown of GALNT2 enhanced malignant phenotypes in gastric cancer cells and promoted tumor metastasis in a nude mouse model. Furthermore, GALNT2 knockdown increases MET activation and reduces Tn antigen expression on MET. These findings suggest that GALNT2 suppresses gastric cancer progression by modifying MET O-glycosylation and phosphorylation.

## MATERIALS AND METHODS

### Real-time reverse transcription (RT)-PCR

Total RNA was isolated from gastric cancer tissues using Trizol reagent (Invitrogen, Life Technologies). Reverse transcription was performed using 2 μg of total RNA, random primers, and SuperScript II RT (Invitrogen) according to the manufacturer's protocol. *GALNT2* expression was quantified by real-time RT-PCR using PCR System Mx3000P (Stratagene) with sense primer 5′-AAGGAGAAGTCGGTGAAGCA-3′ and anti-sense primer 5′-TTGAGCGTGAACTTCCACTG-3′. The other GALNT members were quantified as previously described [6]. Relative mRNA expression normalized to GAPDH was analyzed with MxPro Software (Stratagene).

### Western blot analysis

Total cell lysates from gastric cancer tissue or cultured cells were used. Equal amounts (30 μg) of extracted protein were resolved on SDS-PAGE by electrophoresis, transferred and blocked in TBST (20 mM Tris–HCl, 137 mM NaCl, and 0.1% Tween 20, pH 7.5). The polyvinylidine difluoride membrane was incubated with primary antibody overnight at 4°C, and then with horseradish peroxidase conjugated secondary antibody for 1 hour. The primary antibodies used were GALNT2 (Sigma), GAPDH, β-actin (Santa Cruz Biotechnology, Santa Cruz, CA, USA), MET and Tyr 1234/1235 phospho-MET (cell signaling). Specific bands were detected by enhanced chemilumination detection system (Amersham, Uppsala, Sweden). Protein signals were quantified by optical density ratios using β-actin expression as a control.

### Tissue immunohistochemistry

For immunohistochemical staining, 5-μm sections of the Paraffin-embedded tissue blocks were probed with GALNT2 polyclonal (1:200, Sigma) and MET (1:200, cell signaling) antibodies diluted with 5% BSA/PBS for 16 hours at 4°C, and stained using the Super Sensitive Link-Label immunohistochemistry Detection System (BioGenex). The intensity of GALNT2 staining was quantified by a microscope-based image analysis program (Image Pro Plus; Media Cybemetics, Silver Spring, MD). At least three random fields in each section were examined and analyzed at 100x magnification. A semi-quantitative immunoreactivity scoring (IRS) system [8,9] was applied to assess the immunostaining. Immunostaining intensity (I) was graded as 0 (no staining), 1 (weak staining), 2 (moderate staining), or 3 (strong staining). The percentage of immuno-reactive cells (P) was graded as 0 (none), 1 (<10%), 2 (10–50%), 3 (51–80%), or 4 (>80%). Multiplication of I and P resulted in an IRS ranging from 0 to 12 for each tumor. We used a grouping algorithm (raw scores, low [IRS 0–6] *vs* high [IRS 7–12]) to test the correlation between GALNT2 expression and clinicopathologic features in gastric carcinoma patients.

### Cell culture

Human gastric cancer cell lines MKN28 and MKN74 were kindly provided by Sang-Uk Han (Korea) in 2013. AGS and MKN-45 cells were kindly provided by Min-Chuan Huang (National Taiwan University, Taiwan) in 2010. N87 cells were purchased from Bioresource Collection and Research Center (Hsinchu, Taiwan). GES-1, the immortalized human gastric epithelial mucosa cell line, was kindly provided by Tang-Long Shen (National Taiwan University, Taiwan) in 2014. All cell lines were authenticated by the provider based on morphology, antigen expression, growth, DNA profile, and cytogenetics. These cells were grown in monolayer cultures in T75 cm^2^ flasks or 10-cm culture plates and maintained in RPMI medium with 10% fetal bovine serum, 2% sodium bicarbonate, 2 mM L-Glutamine, and 1% penicillin, 1% streptomycin, and 1% amphotericin at 37°C with 5% CO_2_ in a 95% humidified atmosphere.

### siRNA knockdown of GALNT2 expression

In transient knockdown experiments, two siRNA oligonucleotides against GALNT2 and a non-targeting siRNA control were synthesized by Invitrogen. AGS and MKN28 cells were transfected with siRNA using Lipofectamine RNAiMAX (Invitrogen) with a final concentration of 100 nM.

### Overexpression of GALNT2 in gastric cancer cells

The RT-PCR products of full-length human *GALNT2* (Accession No. NM_004481) were cloned into pcDNA3.1/myc-His (Invitrogen Life Technologies) to generate the *GALNT2*/myc-His fusion gene. The insert was confirmed by DNA sequencing. Overexpression of the *GALNT2* gene was achieved by transfecting AGS (or MKN45) cells with pcDNA3.1/*GALNT2*/mycHis plasmids using Lipofectamine 2000 (Invitrogen, Life Technologies) according to the manufacturer's protocol. The transfected cells were selected with 500 μg/mL of G418 for 14 days and then pooled for further studies.

### Cell viability

The cell viability was assessed by measuring the ability of cells to reduce 3-(4,5-dimethylthiazol-2-yl)-2,5-diphenyltetrazolium bromide (MTT) to the dark blue formazan product. According to the manufacturer's instructions (Cayman Chemical, Ann Arbor, MI), gastric cancer cells were seeded at a density of 1 × 10^3^ cells per well and incubated with MTT for 4 hours at 37°C. Absorbance was read at 570 nm. Results are expressed as percent absorbance compared to the control cells.

### Transwell migration assay

Cell migration was evaluated in 24-well transwell culture chambers. The siGALNT2-transfected cells (5 × 10^3^) were re-suspended in serum-free RPMI and added to the upper well of each migration chamber with an 8-μm pore size membrane (Corning). Cell migration was induced by 10% FBS (PAA Laboratories) in the lower chamber. After 24 hours, cells that migrated to the lower surface of the filter were stained with 0.5 % (wt/vol) crystal violet (Sigma) and counted.

### Matrigel invasion assay

Cell invasion assays were done in BioCoat Matrigel invasion chambers (Becton Dickinson) according to the manufacture's protocol. Briefly, RPMI with 10% FBS as a chemoattractant was loaded in the lower part of the chamber, and 5 × 10^3^ transfected cells in 500 μL serum-free RPMI were seeded onto the upper part. Cells were allowed to invade the matrigel for 24 hours. Invading cells were fixed and stained with 0.5% (wt/vol) crystal violet. Cell numbers were counted for each well, and values are presented as mean ± SD.

### Plasmids and construction of stable transfectants

To stably knock down endogenous GALNT2 in AGS cells, *GALNT2* shRNA plasmids (pGPU6/GFP/Neo-shGALNT2) were designed and synthesized by Tools Co. (Taipei, Taiwan). The target sequence was 5′-GATGGTGTGGTTGGAGTTTATGAAT-3′. The pGPU6/GFP/Neo-shC plasmid, which encodes a hairpin siRNA with a sequence not found in human genome databases, was used as a negative control [10]. AGS cells were transfected with plasmids expressing shGALNT2 and shC using Lipofectamine 2000 (Invitrogen) according to the manufacturer's instructions. Stable AGS transfectants were confirmed by Western blotting. The stable transfectants expressing *GALNT2*-specific shRNA are referred to as shG2, and control AGS cells transfected with shC are referred to as shC.

### Tumor metastasis in nude mice

Four week-old male nude *Balb/c* mice (National Taiwan University, Taipei, Taiwan) were used to assess the effect of GALNT2 shRNA on tumor metastasis *in vivo*. The protocol was approved by the Animal Care and Use Committee of National Taiwan University. The mice were divided to two groups, with nine per group. Approximately 2 × 10^6^ cells (AGS shC or AGS shGALNT2) in 0.1 ml of PBS were inoculated into the tail veins of the mice [10]. Tumor–bearing mice were monitored by the *in vivo* imaging system (IVIS) (IVIS spectrum, Xenogen Corporation, CA, USA) to assess the metastasis of gastric cancer cells. At indicated times, mice were anesthetized and placed in the IVIS to analyze fluorescence according to the manufacturer's protocol. Quantitative measurement of fluorescence signal intensity was performed using Living Image v.4.1 software (PerkinElmer/Caliper) [11].

### Phospho-receptor tyrosine kinase array

A human phospho-receptor tyrosine kinase (p-RTK) array kit including 49 RTKs was purchased from R&D systems. siC and siGALNT2-transfected gastric cancer cells (AGS and MKN28) were cultured until confluence on 10 cm^2^ culture plates. Cells were lysed and 250 μg of protein were used for Western blotting according to the manufacturer's protocol. Activated receptors were matched according to the phospho-RTK array coordinates: (b1, b2: EGFR; b17, b18: INSR; b19, b20: IGF1R; c3, c4: MET; Black dots represent phospho-tyrosine positive controls.)

### Lectin pull down assay

*Vicia Villosa* Lectin (VVA) agarose beads (Vector Laboratories) were used to detect the Tn antigen on glycoproteins, as reported [12]. Cell lysates (0.3 mg) were incubated with or without neuraminidase, an enzyme that removes sialic acids, at 37°C for 1 hour, and then applied to VVA-conjugated agarose beads at 4°C for 16 hours. Precipitated proteins were then used for Western blotting.

### Gene expression profiling and quantitative RT-PCR validation

Total RNA from control and GALNT2 siRNA knockdown AGS cells was extracted in triplicate using the GeneJET RNA Purification kit (Thermo Scientific) following the manufacturer's protocol and quantified by NanoDrop spectrophotometer (Bio-Rad). The RNA quality was monitored with an Agilent 2100 Bioanalyzer (Agilent Technologies, Santa Clara, CA). cDNA prepared from 10 μg of total RNA was labeled with aa-dUTP using the Invitrogen SuperScriptTM Plus Indirect cDNA Labeling System according to the manufacturer's protocol, followed by aa-cDNA column purification (QIAGEN, Valencia, CA). Alexa555 Dye was incorporated to aa-cDNA followed by column purification with Alexa/CyDye-cDNA cRNA purification (Qiagen). DNA yields were confirmed by 1% DNA agarose gel and visualized with Fuji image reader at 600V PMT. An Agilent Gene Expression Hybridization Kit was used for hybridization according to the manufacturer's instructions. Briefly, 16 μl of dye-labeled cDNA were hybridized to an Agilent SurePrint G3 Human Gene Expression 8x60K v2 Microarray (G4851B). Microarrays were scanned on an Agilent DNA Microarray Scanner (US9230696) using one color scan setting for 8x60k array slides. The scanned images were analyzed with Feature Extraction Software 10.5.1.1 (Agilent). Features flagged in Feature Extraction as Feature Non-uniform outliers were excluded. Quantitative RT-PCR to determine expression of selected genes was performed for validation as described above. The primers were designed with Primer3 (v.0.4.0) algorithm; the sequences are freely available from the Entrez Nucleotide database. All microarray experiments were performed in triplicate, with three hybridizations conducted for each group of *GALNT2* knockdown cells against the corresponding control. Microarray data were deposited in the GEO database (accession number GSE75755).

### Statistical analyses

We conducted paired *t*-tests for the analysis of paired GC tissues. *In vitro* tumor cell viability migration and invasion data were analyzed by one-way analysis of variance (ANOVA). Disease-free survival data were analyzed by Kaplan-Meier log rank tests. Student's *t*-test was used for other experiments. Data are presented as means ± SD. *p*<0.05 or less was considered to be statistically significant, and all experiments were performed in triplicate to verify reproducibility.

## SUPPLEMENTARY FIGURES AND TABLE




