# Supplementary material for: Mucin glycosylating enzyme GALNT2 suppresses malignancy in gastric adenocarcinoma by reducing MET phosphorylation
Source: Oncotarget. 2016 Jan 30;7(10):11251–62. doi: 10.18632/oncotarget.7081 (PMC4905470; doi:10.18632/oncotarget.7081)
Supplement: Supplementary file 1 [file oncotarget-07-11251-s001.pdf]

SUPPLEMENTARY FIGURES AND TABLE

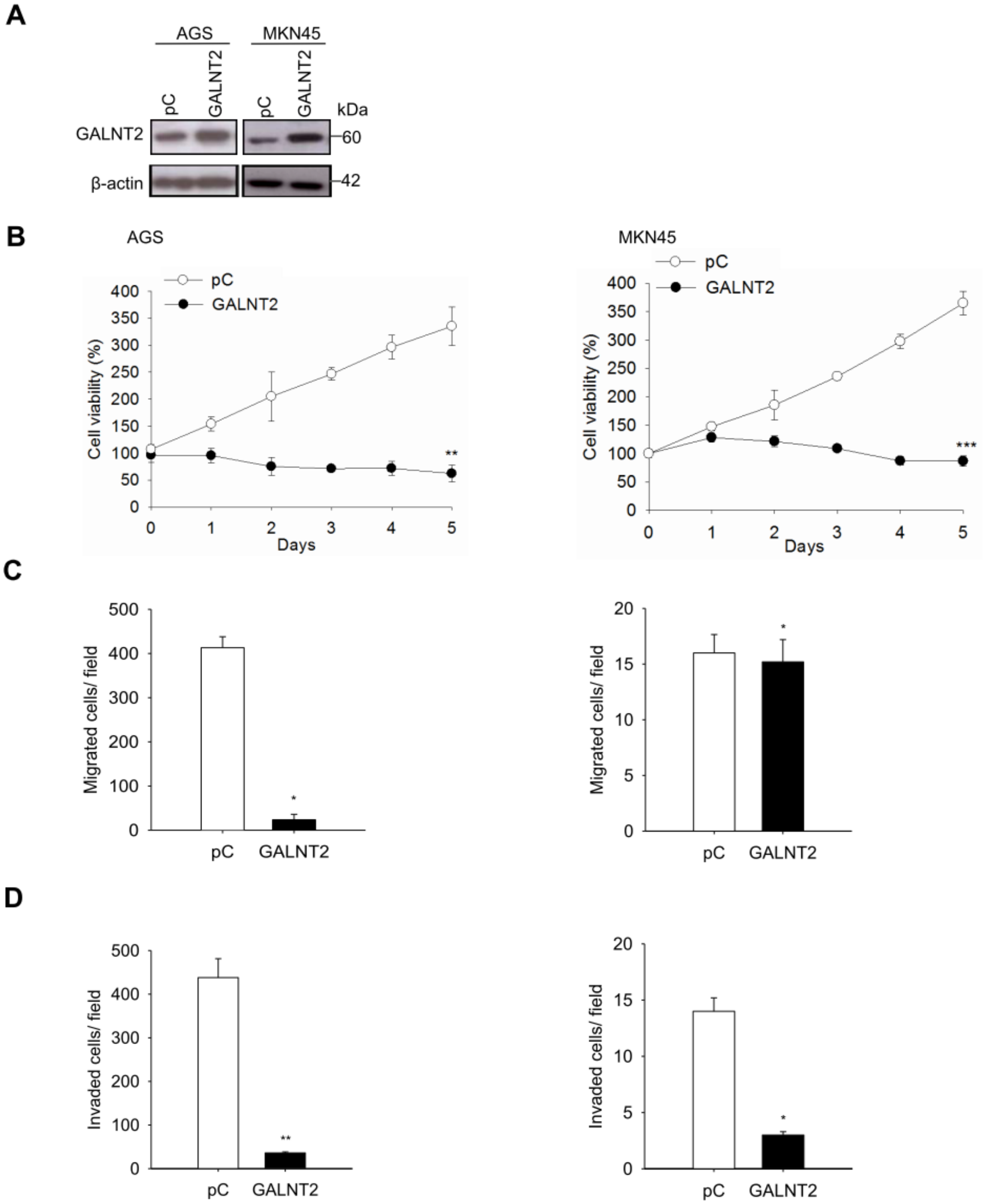

Supplementary Figure S1: Overexpression of GALNT2 in gastric cancer cell lines and its effect on phenotypes. **A.** Overexpression GALNT2 in AGS and MKN45 cells was confirmed by Western blotting. **B.** Cell viability was analyzed by MTT assay at different time points. The results were graphed after standardization by GALNT2 in control cells (pC) (Day0) to 1.0. (Left, AGS cells; right, MKN45 cells) **C.** Effects of GALNT2 overexpression on cell migration by transwell migration assays. (Left, AGS cells; right, MKN45 cells) **D.** Effects of GALNT2 overexpression on cell invasion by matrigel invasion assay. (Left, AGS cells; right, MKN45 cells) (pC: plasmid control; *:$p < 0.05$;**:$p < 0.005$; ***:$p < 0.001$).

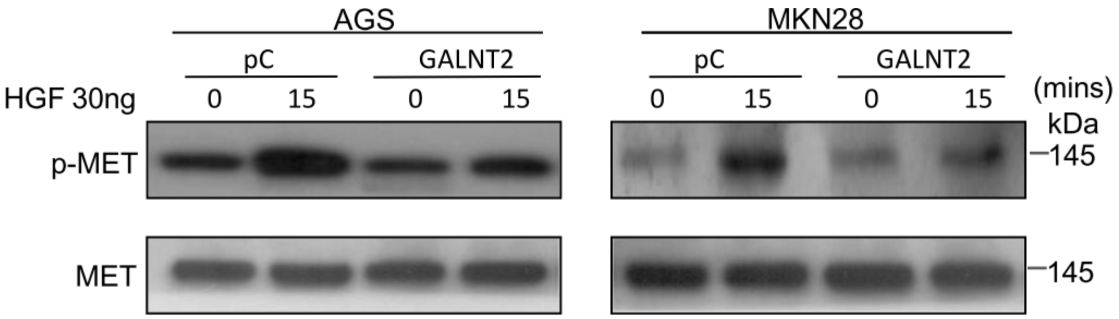

Supplementary Figure S2: Overexpression of GALNT2 in gastric cancer cells reduces HGF-induced p-MET. AGS and MKN28 cells were transfected with plasmid control (pC) or pcDNA3.1/*GALNT2*/mycHis plasmids. The cell lysates were immunoblotted with antibodies for p-MET and total MET. A marked decrease in MET phosphorylation in both AGS and MKN28 cells was evident at 15 minutes after HGF stimulation.

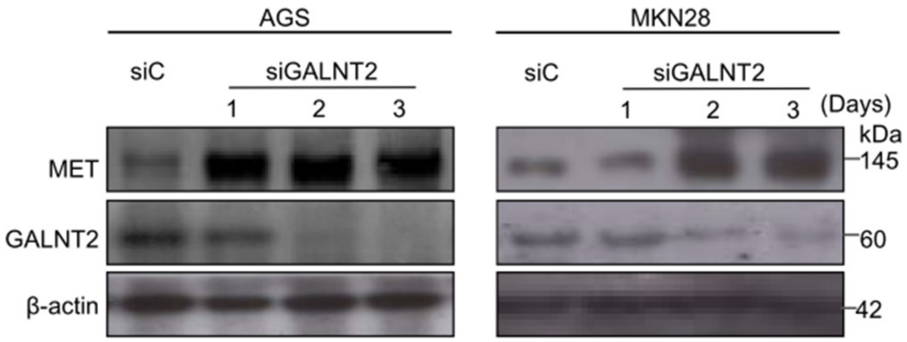

Supplementary Figure S3: Western blots of GALNT2 and total MET expression in AGS and MKN28 cell lines. Total MET expression in AGS and MKN28 were increased when GALNT2 was knockdowned.

Supplementary Table S1: Differential expressions of selected genes in GALNT2 knockdown AGS cells

See Supplementary File 1
